# Neural Network-Based Active Load-Sensing Scheme and Stiffness Adjustment for Pneumatic Soft Actuators for Minimally Invasive Surgery Support

**DOI:** 10.3390/s23020833

**Published:** 2023-01-11

**Authors:** Yuxi Lu, Zhongchao Zhou, Shota Kokubu, Ruian Qin, Pablo E. Tortós Vinocour, Wenwei Yu

**Affiliations:** 1 Department of Medical Engineering, Graduate School of Engineering, Chiba University, Chiba 263-8522, Japan; 2 Center for Frontier Medical Engineering, Chiba University, Chiba 263-8522, Japan

**Keywords:** NN-based active load-sensing scheme, exploration strategy, soft actuator in MIS, adjust stiffness

## Abstract

To provide a stable surgical view in Minimally Invasive Surgery (MIS), it is necessary for a flexible endoscope applied in MIS to have adjustable stiffness to resist different external loads from surrounding organs and tissues. Pneumatic soft actuators are expected to fulfill this role, since they could feed the endoscope with an internal access channel and adjust their stiffness via an antagonistic mechanism. For that purpose, it is essential to estimate the external load. In this study, we proposed a neural network (NN)-based active load-sensing scheme and stiffness adjustment for a soft actuator for MIS support with antagonistic chambers for three degrees of freedom (DoFs) of control. To deal with the influence of the nonlinearity of the soft actuating system and uncertainty of the interaction between the soft actuator and its environment, an environment exploration strategy was studied for improving the robustness of sensing. Moreover, a NN-based inverse dynamics model for controlling the stiffness of the soft actuator with different flexible endoscopes was proposed too. The results showed that the exploration strategy with different sequence lengths improved the estimation accuracy of external loads in different conditions. The proposed method for external load exploration and inverse dynamics model could be used for in-depth studies of stiffness control of soft actuators for MIS support.

## 1. Introduction

In Minimally Invasive Surgery (MIS), rigid or soft surgical instruments need to be inserted into human body through incisions of minimal size or natural orifices for accessing the target sites. Compared to traditional open surgery which requires large incisions, MIS has been found to provide patients with a variety of benefits, including improved safety, cosmesis, shorter recovery time, shorter hospital stay, fewer postoperative complications, and less pain from the patient’s point of view [1]. Consequently, MIS has gradually supplanted traditional open surgery in recent years [2]. The surgical view in MIS is provided by endoscopes. However, the workspace of MIS is often small, easily deformable, dynamically changing and results in limiting the movements of endoscope. In certain types of MIS, the position where the endoscope is inserted into the body acts as a pivot point, reversing the surgeon’s motion. This fulcrum effect prevents surgeons from using lengthy, rigid endoscopes to observe the specific MIS procedure area of interest [3].

In addition, some companies such as Ambu Inc. and Olympus Corp have already manufactured flexible endoscopes, which consists of a long flexible tube with a high-resolution camera and a light source at its tip. Their tips have a single degree of freedom (DoF) and can be controlled by means of a one thumb-controlled toggle or two thumb-controlled dials at their end. They can vary in diameter and length depending on the application and the patient. Flexible endoscopes are usually applied in thoracoabdominal and bladder-related surgical procedures [4,5,6]. 

Notably, the flexible endoscope is susceptible to external load from surrounding organs or tissues, especially when it must traverse or pass through several organs during a surgical procedure, such as a Transvesical Prostatectomy [7]. In MIS procedures, it is necessary to adjust an appropriate stiffness of the flexible endoscope to resist the different external loads from surrounding organs and tissues and provide a stable surgical view for surgeons. When the stiffness of flexible endoscope is not enough, the external load could make that the flexible endoscope very susceptible to deformation and bending. The deformation and bending of endoscope can make the surgical view unstable and cause surgeon to spend a lot of extra time to adjust the position of endoscope. On the other hand, the excessive stiffness of flexible endoscope may raise the risk of the damage to the surrounding organs or tissues. It also could limit the movement and bending of the flexible endoscope, cause a reduction of the surgical view. However, the flexible endoscope cannot adjust its own stiffness. Therefore, it is necessary to design an instrument to change the endoscope’s stiffness.

Soft actuators can self-adjust their stiffness and are ideal for dealing with unstructured environments or interacting with humans because their softness allows them to deform around their surroundings. Soft actuators are also commonly employed in different fields, such as rehabilitation [8,9,10,11,12,13] and MIS instruments [14,15]. In numerous applications, a pneumatic soft actuator is expected to have a small size, low hardness, large bidirectional bending angle and low stiffness, hence, minimizing the damage of surgical instruments to the surrounding tissues. Thanks to an internal access port of the soft actuator body, some of them could be utilized to feed endoscopes or other MIS instruments [15,16].

The first type of pneumatic soft actuators could self-adjust stiffness rely on internal Shape Memory Alloys (SMA) [17] or a granular jamming-based stiffening mechanism [18]. The SMA-based pneumatic soft actuator could adjust the stiffness of the flexible endoscope while it is fed. Variable stiffness could enable the flexible endoscope to resist loads from surrounding organs or tissues and enhance its operability, providing a stable surgical view for MIS procedures. Although SMA or granular material might provide sufficient stiffness, their bulk and weight prevent pneumatic soft actuators from being miniaturized and lightweight. In addition, SMAs require heat or electricity to increase their stiffness, which diminishes their safety and applicability in MIS. The second form of pneumatic soft actuator may modify its own stiffness via an antagonistic mechanism [19]. This kind of pneumatic soft actuator can vary its own stiffness by altering the air pressure against the opposing air chamber without additional components. However, when the endoscope is inserted in a pneumatic soft actuator, the endoscope could affect stiffness adjustment using the antagonistic mechanism due to internal interference between the pneumatic soft actuator and endoscope.

In some cases, when a pneumatic soft actuator with an endoscope is compressed and deformed by an external load or resistance from the surrounding organs and tissues, the pneumatic soft actuator should increase stiffness to resist the load. However, an over-stiffness generated by high air pressure to both antagonistic chambers may raise the risk of not only damage to the surrounding organs, but also air leakage from the soft actuator [6]. Therefore, to select an appropriate stiffness, the external load should be estimated [17,18]. However, conventional load sensors have proven inappropriate for pneumatic soft actuators since directly embedding them affects the omnidirectional compliance of the pneumatic soft actuator [20]. Alternatively, soft sensors made of soft materials have also attracted the interest of a number of researchers. Soft sensors are easy to integrate with soft actuators in a range of applications due to their high compliance [21,22,23]. This kind of soft-load sensor measures and models changes in electrical properties, such as resistance or capacitance in response to material stress and strain changes. However, the manufacturing of these actuator–sensor combinations is extraordinarily challenging. Incorrect sensor arrangement might easily damage the actuator and cause it to leak [20]. In addition, the other soft sensors are typically limited to certain settings, such as those containing magnetic or bright light sources [24,25].

In recent years, there has been increasing interest in soft actuators embedded with sensing mechanisms [26,27,28,29]. In these soft actuators, sensing layers or units such as fiber embroidery [26], thin biphasic metal films [27], conductive working fluids [28] or wires [29] were structured within the body of the soft actuators. The analytical models for monitoring the actuator’s movement (or load) were deduced based on mechanical or electrical principles. For example, a soft pneumatic actuator with self-sensing of its external load was reported in [27]. They embedded a sensing layer in soft actuator which is difficult to be fabricated. Its sensing mechanism was based on an analytical model of relative change in resistance and load. Due to its high sensitivity, the sensing mechanism was suitable for measuring dynamically changing signals such as vibration or impact force. However, it is impossible to measure static external load which is needed to decide the stiffness. In addition, accurate analytical models underlying the self-sensing pose another big challenge when applied to MIS. That is, in the MIS support scenario, when different flexible endoscopes are inserted into a pneumatic soft actuator, the characteristics of the soft actuator (bending, stiffness, deformation) change, thus the analytic model is also altered. Therefore, the application of a model-based self-sensing in MIS support is complicated by the need to adjust the analytical model. Moreover, when the flexible endoscope is inserted into a pneumatic soft actuator and bent by it, there is an internal interference between flexible endoscope and pneumatic soft actuator. The internal interference is very complex and, consequently, it also influences the effect of the flexible endoscope on the characteristics of the pneumatic soft actuator complex. For example, the overall stiffness of the pneumatic soft actuator and flexible endoscope could be changed with the bending angle and this relationship is nonlinear. Therefore, it is also a challenge to make an accurate analytical model for the pneumatic soft actuator when inserted with the flexible endoscope. Due to these challenges to the self-sensing-based analytical model, it is necessary to use neural networks (NNs) to adaptively acquire the model, which have been effectively applied for modeling the nonlinearity and uncertainty [30,31].

Self-sensing approaches based on NNs have been proposed, as NNs are capable of approximating functions with great precision. The defining characteristic of this method is that sensing is realized with neither independent nor embedded physical sensors; instead, it makes use of the relationship between target physical quantities and a set of state variables that are directly or indirectly affected by or interacting with the physical quantities. The relationship can be learned as a function from collected raw data of a soft actuator [30]. A NN-based bending angle and contact force self-sensing was proposed and implemented for a 3D-printed pneumatic soft actuator [20]. After learning, the NN could predict the bending angle and contact force using air pressure values. Although the NN could accurately predict for the case similar to the samples in the training dataset, for the real test samples, it generated highly deviant predictions. Rather than the learning capability of the NNs, the influence of the nonlinearity of the soft actuating system and uncertainty of interaction between the soft actuator and its environment were the major reasons [31]. This is not surprising, as real-world robotic application suffers from the same problem [30].

In this study, we proposed a NN-based active load-sensing scheme and stiffness adjustment for a pneumatic soft actuator for MIS support with antagonistic chambers for three degrees of freedom (DoFs) of control (described as soft actuator for simplicity). An environment exploration strategy was studied for improving the robustness of the sensing. The minimal exploration sequence was determined by the estimation error and change in the smallest bending angle. A NN-based inverse dynamics model was established for estimating the air pressure from the predicted load and desired stiffness.

This paper is organized as follows. Section 2 (Materials and Methods) introduces the experimental setting for collecting, training and testing datasets, and the NNs for the active-sensing scheme of external load and controlling air pressure to realize desired stiffness. Section 3 (Results) shows the results of preliminary experiments, the active load-sensing scheme and the NN-based inverse dynamics model for stiffness. Section 4 (Discussion) gives the discussion about the results from Section 3 and Section 5 includes the conclusion and future work for active load-sensing scheme for soft actuators with multi-DoFs and control of stiffness.

## 2. Materials and Methods

### 2.1. Measurement of Bending Angle and Stiffness for the Soft Actuator

**Figure 1:**
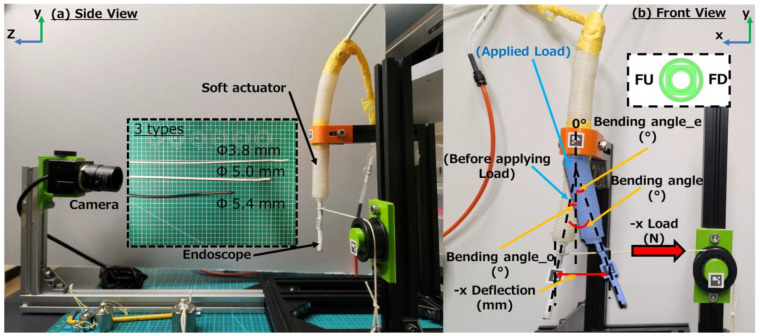
Measurement method of soft actuator (**a**) side view, (**b**) front view.

**Figure A1:**
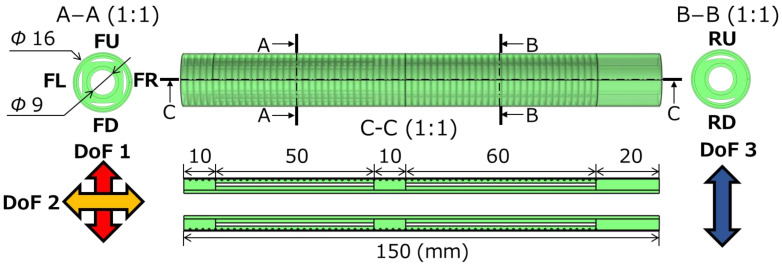
Investigated three-DoFs pneumatic soft actuator designs and parameters.

The measurement approach for the soft actuator prototype experiments is depicted in Figure 1. The design and fabrication process of the soft actuator is described in Appendix A. There are two DoFs in the front of the soft actuator. DoF 1 consisted of a front up chamber (FU, Figure A1) and front down chamber (FD, Figure A1). DoF 2 consisted of a front left chamber (FL, Figure A1) and front right chamber (FR, Figure A1). DoF 1 and DoF 2 have the identical bending characteristics and structure except for their orientation. For example, when inflating the FU and FD with 10 kPa and 100 kPa air pressure, respectively, the soft actuator bends 41.4 degree in the x direction (refer to Figure 1) of DoF 1. Meanwhile, when inflating the FL and FR with 10 kPa and 100 kPa air pressure, respectively, the soft actuator also bends 41.4 degree in DoF 2. In our work, DoF 1 (FU and FD) was chosen for investigation. Due to the symmetry of two chambers (FU and FD), the asymmetric air pressure for them can generate the movement for the respective bending direction. Therefore, one bending direction was measured. The air pressure for FU (P_FU) varied between 0 and 30 kPa. The air pressure of FD (P_FD) has a range of 0–110 kPa. For each 10 kPa increase in pressure on either side, the bending angle and stiffness were recorded once, resulting in a total of 48 distinct data samples.

As shown in Figure 1, before applying an external load, an endoscope was inserted in the soft actuator and the bending angle of the soft actuator was measured as bending angle_original (bending angle_o). After all the chambers were inflated to a steady state, the endoscope and soft actuator were bent by applying various external loads (0 N, 0.15 N, 0.17 N, 0.22 N, 0.27 N, 0.56 N, 1.06 N). Afterwards, the endoscope was stabilized again and at a certain angle which was recorded as bending angle_end (bending angle_e). The horizontal displacement endoscope was recorded as -x deformation. All the coordinate information was measured by ArUco markers (2D makers, [32]) which were set in front of all soft actuators (squared binary marker). The markers were photographed through a camera (USB8MP02G-SFV, ELP camera, Ailipu Technology Co., Ltd., Shenzhen, China) during the bending process and OpenCV with Python 3.8.12 was used for the calculation of the soft actuator’s bending angle_o, bending angle_e and -x deformation.

Because endoscopes of varying diameters impede the expansion of the soft actuator’s chamber differently, the bending angle_o, bending angle_e, and -x deformation were all measured with the soft actuator inserted in three different types of endoscopes (Ambu^®^ aScopeTM 4 Broncho Slim, diameter: 3.8 mm; Ambu^®^ aScopeTM 4 Broncho Regular, diameter: 5.0 mm; Ambu^®^ aScopeTM 4 Broncho Cysto, diameter: 5.4 mm; Ambu Inc., Copenhagen, Denmark, displayed in Figure 1 left). The stiffness of the 5.4 mm endoscope, which is used in bladder surgery, was lower than those of the 3.8 mm and 5.0 mm endoscopes, which used in trachea surgery.

In an MIS procedure, the flexible endoscope’s lens should be kept a distance (axial direction) from organs or tissues to provide a surgical view for surgeons. Therefore, the external load in the axial direction was very small, which allowed us to focus on the external load in the horizontal direction. Adjusting the stiffness on −x direction could help the flexible endoscope to resist different external loads in the −x direction. The overall stiffness in the −x direction (K) of the soft actuator and endoscope is calculated by Equation (1). The K cannot contain the stiffness in the other direction such as axial direction (−y direction). The load of the −x direction is the partial load in the horizontal direction calculated by the angle between the rope and the horizontal direction.

(1) $$K=\frac{-x\;Load}{-x\;Deflection}$$

−x Load: load for −x direction; −x Deflection: deflection after applying load for −x direction.

### 2.2. A Active Load-Sensing Scheme and Environment Exploration Strategy

As stated in the Section 1, it is necessary to estimate the external load for selecting the appropriate soft actuator’s stiffness, an active load-sensing scheme is proposed in this paper.

It is considered that, the sufficient and necessary set of input features of the NN is P_FU, P_FD, bending angle_o, bending angle_e.

First, the inflation of antagonistic chambers may modify the stiffness. Although the modification is angle-dependent, it is a preferrable characteristic since the adjustable stiffness can be used to deal with different situations, including different endoscopes and external load. Meanwhile, the angle of the soft actuator may also be changed by the inflation of antagonistic chambers.

Second, as in the other self-sensing studies [20,30], the air pressure to the chambers causes bending of the soft actuator as its effect. This cause-and-effect is affected by the stiffness of the complex of the soft actuator body and inserted endoscope, and external load. Although the stiffness of soft actuator is angle-dependent, it is not a free variable, thus, the external load is the determining factor.

For these two reasons, both the pressure to the antagonistic chambers and the angles before and after applying external load are necessary input features. However, these input features are not sufficient for predicting the external load, since they are affected by the combined effect of the overall stiffness of the soft actuator and endoscope, and the external load. This combined effect can be further decoupled in the inverse dynamics model for realizing desired stiffness as described in Section 2.3, by using the information from the endoscope.

**Figure 2:**
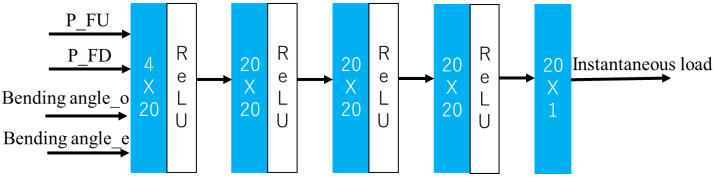
The architecture of the active load-sensing scheme.

Figure 2 illustrates the architecture of the active load-sensing scheme. The input data shape is (1,4). The three hidden layers each have 20 nodes and output layer contains one node representing the instantaneous load. The activation function of the scheme is ReLu. The active load-sensing schemes were developed with Python 3.9.12 64-bits, PyTorch “1.12.1” with cuda “11.7”. The optimizer is the Adam optimizer with learning rate = 1 × 10^−5^, and the total epochs are 8 × 10^4^. The training data consists of all data with external loads of 0, 0.17, 0.27, 0.56 and 1.06 N for endoscope diameter 0, 3.8 and 5.4 mm, while the verification data consists of all data with external loads of 0.15 and 0.22 N for 5 mm endoscopes. An output with 10% deviation from its ground-truth external load is defined as a successful prediction.

An environment exploration strategy was studied for improving the robustness of the sensing. The minimal exploration sequence was determined by the prediction error. After the network estimates the instantaneous load based on the current state (current P_FU, P_FD, bending angle_o, bending angle_e), a sequence of actuation is implemented to explore the environment for dealing with the influence of the nonlinearity of the soft actuating system and the uncertainty of interaction between the soft actuator and its environment. Taking FD as an example, P_FD(n) is calculated by Equation (2).

(2) P_FD(n)=current P_FD+n×m_step

A positive value of *n* means forward exploration (e.g., +1, +2…), while a negative value means backward exploration (e.g., −1, −2), and m_step is a minimal exploration step (10 kPa in this study). Six different exploration sequences (“+1, +2”, “+1, +2, +3”, “+1, +2, +3, +4”, “−1, +1”, “−1, −2, +1, +2”, “−1, −2, −3, +1, +2, +3”) were investigated. The biggest step number of exploration sequence is 6 and the smallest step number of exploration sequence is 2. The average of all the predicted external load values of all the steps in a sequence was used as the external load of the sequence.

### 2.3. A NN-Based Inverse Dynamics Model for Stiffness

In addition to the estimated external load, desired K, bending angle_e and P_FU, the diameter of the endoscope can affect the overall stiffness of the soft actuator and endoscope, so it was also added to the input of the network. The appropriate stiffness was selected according to the external load. Therefore, the stiffness calculated from P_FU, P_FD, endoscope diameter and bending angle_e and external load can be referred to as a forward dynamic model for stiffness. Moreover, when a desired stiffness was provided, the function which can calculate the P_FD is a complex and nonlinear inverse dynamic model for stiffness and is difficult be modelled. NNs are always used in solving the learn inverse dynamic model [26]. To control the soft actuator to a desired stiffness, we trained a multilayer perceptron (MLP) to represent an inverse dynamics model for stiffness. Multilayer perceptron (MLP) is a fully connected class of feedforward artificial neural network (ANN) [31]. Desired K, external load, bending angle_e and diameter of the endoscope were set as input.

**Figure 3:**
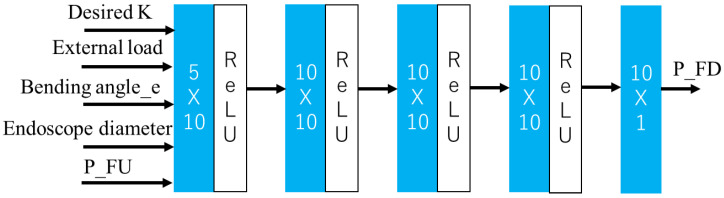
The architecture of NN-based inverse dynamic model.

Figure 3 illustrates the architecture of the model, which was also developed with Python 3.9.12 64-bits. The activation function is ReLu, the optimizer was the Adam optimizer with learning rate = 1 × 10^−3^, and the total epochs are 5 × 10^4^. This section employed the same data set as the active load-sensing scheme section for both the training and verification data; however, an extra target stiffness, external load and endoscope diameter value are included in the input data of model, and the desired stiffness of both sets was calculated according to Equation (1). At the same time, we removed bending angle_e from the input data of the model.

## 3. Results

### 3.1. Preliminary Result—Bending Angle_o of Soft Actuator before Applying External Load

**Figure 4:**
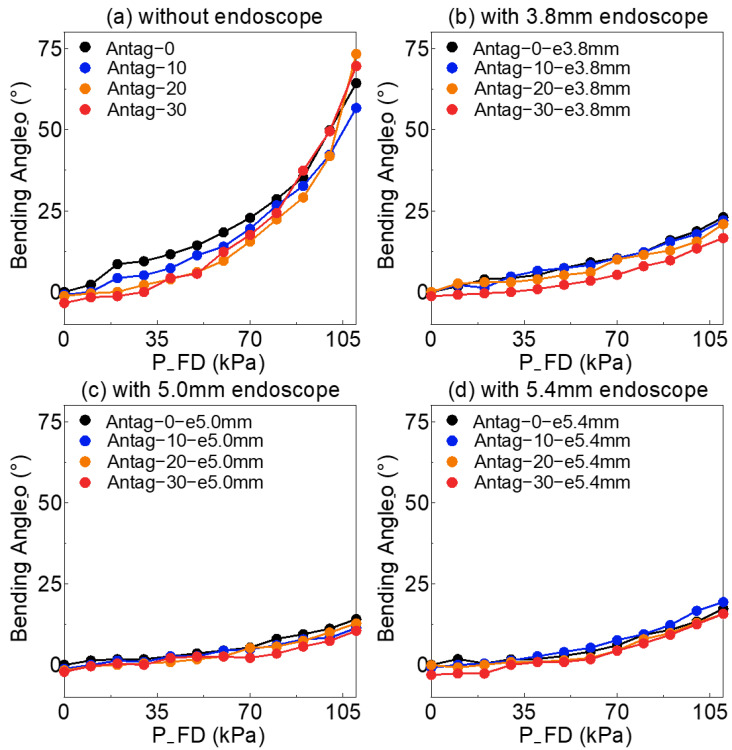
The variation of soft actuator’s bending angle before applying external load under different sets of FU and FD air pressure (**a**) without endoscope: without inserting endoscope into soft actuator for experiment, (**b**) with 3.8 mm endoscope: inserting 3.8 mm endoscope into soft actuator for experiment, (**c**) with 5.0 mm endoscope: inserting 5.0 mm endoscope into soft actuator for experiment, (**d**) with 5.4 mm endoscope: inserting 5.4 mm endoscope into soft actuator for experiment.

Figure 4a shows the bending angle_o of the soft actuator without an endoscope, under a different set of agonists (P_FD) in the *x*-axis (range 0–110 kPa) and antagonist (P_FU) which was the legend by Antag-0, 10, 20 and 30. Figure 4b–d shows the bending angle_o of the soft actuator with a 3.8, 5.0 and 5.4 mm endoscope, respectively.

As shown, when P_FU was constant, the bending angle_o increased as P_FD increased, and the rate of increase of the bending angle_o increase more rapidly from 70 kPa. In contrast, when P_FD was held constant, a greater P_FU reduced the bending angle_o, indicating that the P_FU had a negative influence on the bending angle_o. Although the angle of Antag-30 was greater than that of Antag-0, 10 and 20 at 70–110 kPa in Figure 4a, the other P_FUs might greatly lower the bending angle. Additionally, the diameter of the endoscope (e3.8 mm, e5.0 mm, e5.4 mm) could significantly affect the bending angle_o, with a larger diameter endoscope having a stronger influence on the bending angle_o, as shown by the comparison of bending angle_o of Antag-0 (67.3°) and Antag-0-e5.4 mm (17.3°) at 110 kPa.

### 3.2. Prototype Experiments for Stiffness of Soft Actuator (K)

**Figure 5:**
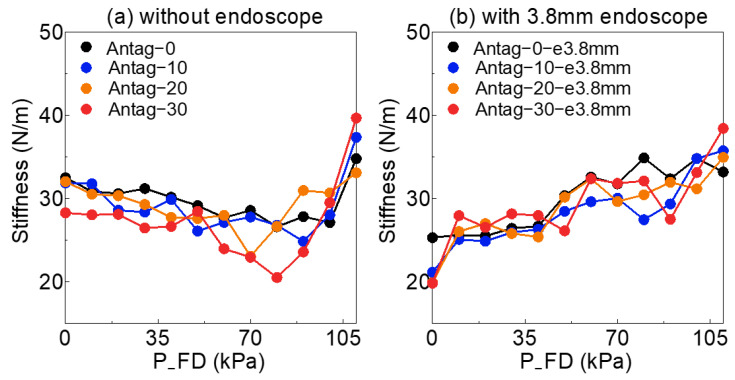

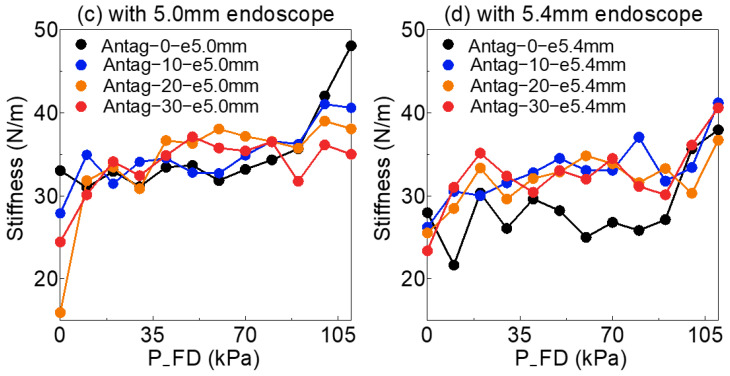
The variation of soft actuator’s stiffness under different sets of FU air pressure and FD air pressure (**a**) without endoscope: without inserting endoscope into soft actuator for experiment, (**b**) with 3.8 mm endoscope: inserting 3.8 mm endoscope into soft actuator for experiment, (**c**) with 5.0 mm endoscope: inserting 5.0 mm endoscope into soft actuator for experiment, (**d**) with 5.4 mm endoscope: inserting 5.4 mm endoscope into soft actuator for experiment.

Figure 5a showed that the soft actuator’s stiffness under different sets of agonists (P_FD) in the *x*-axis (range 0–110 kPa) and antagonist (P_FU) which was the legend by Antag-0, 10, 20 and 30 without an endoscope. Figure 5b–d shows the total stiffness of the soft actuator with a 3.8, 5.0 and 5.4 mm endoscope, respectively.

Figure 5 indicated that the same P_FU had different effects on stiffness under the different P_FD. On the other hand, as the P_FD exceeded 90 kPa, the stiffness of the soft actuator almost increased as P_FD increased. P_FU was also shown to have a non-linear effect on the soft actuator’s stiffness. When air pressure was less than 90 kPa, this influence was different from expected, particularly in the absence of an endoscope, and P_FD reduced the stiffness of the soft actuator (under 90 kPa).

**Figure 6:**
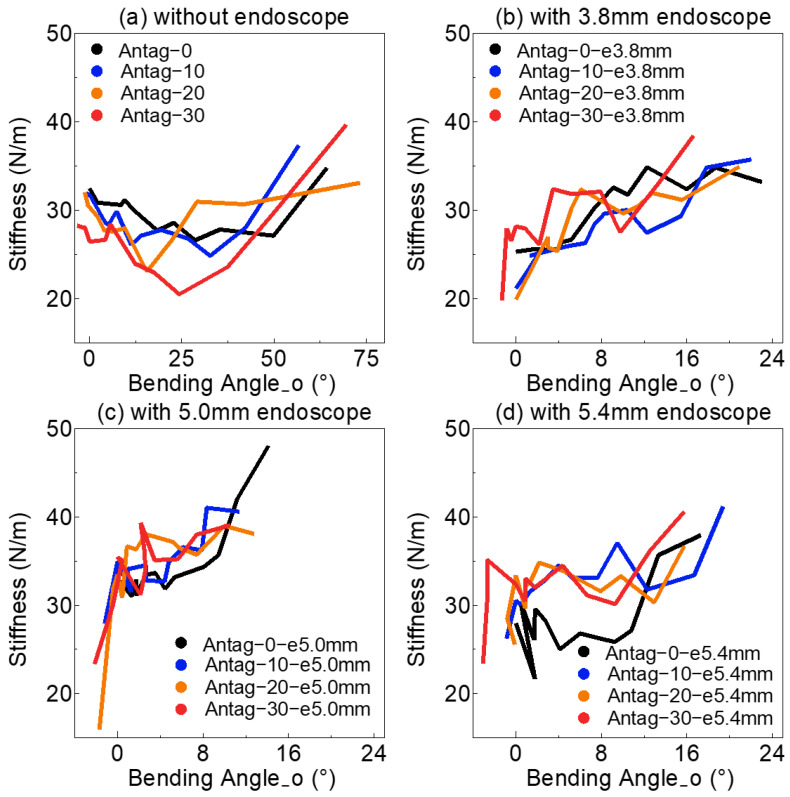
The variation of soft actuator’s stiffness under different bending angle_o (**a**) without endoscope: without inserting endoscope into soft actuator for experiment, (**b**) with 3.8 mm endoscope: inserting 3.8 mm endoscope into soft actuator for experiment, (**c**) with 5.0 mm endoscope: inserting 5.0 mm endoscope into soft actuator for experiment, (**d**) with 5.4 mm endoscope: inserting 5.4 mm endoscope into soft actuator for experiment.

In addition, various sets of P_FU and P_FD resulted in varied bending angle_o values, which had significant influences on the soft actuator’s internal structure and further affected the stiffness of the soft actuator. Figure 6 depicts the relationship between the bending angle_o and soft actuator’s stiffness. As shown, the stiffness of the soft actuator with the three types of endoscopes was all greater than 30 N/m at a considerable bending angle_o (above 8°). Even when the stiffness of the actuator without the endoscope did not reach 30 N/m, a gradual increase in actuator stiffness occurred for bending angle_o greater than 30°.

### 3.3. The Results of Active Load-Sensing Scheme and Environment Exploration Strategy

**Figure 7:**
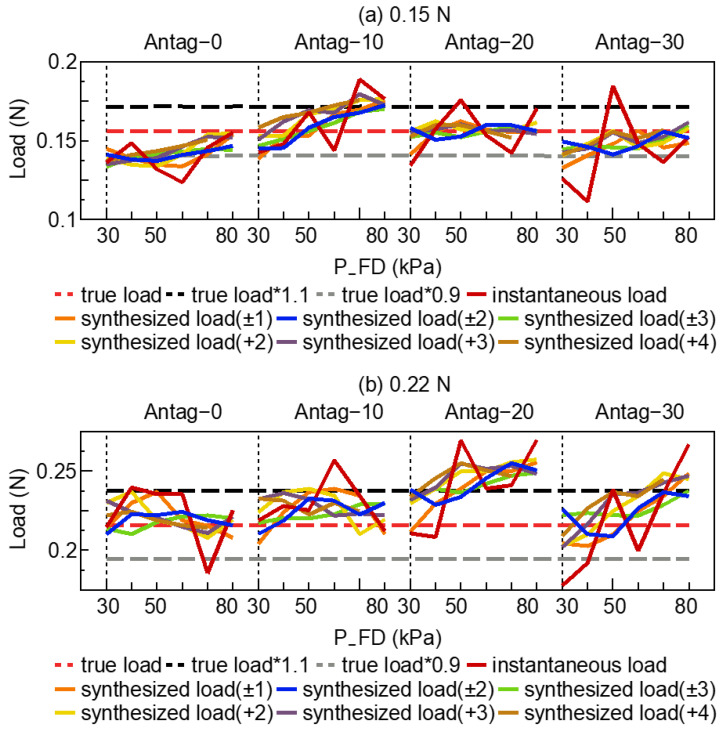
The test data of active load-sensing scheme with 5.0 mm endoscope from NN (**a**) 0.15 N, (**b**) 0.22 N.

Figure 7 depicts the results of the active load-sensing scheme, where the true loads in Figure 7a,b were 0.15 N and 0.22 N, respectively. The mean and variance of the different exploration sequences are shown in Table 1 (for 0.15 N) and Table 2 (for 0.22 N).

The instantaneous load was the output of a NN that was only based on the present state and legend by instantaneous load. The synthesized estimation of external load with the different exploration strategies are the legend on the synthesized load (±n or +n). Figure 7, Table 1 and Table 2 demonstrate that, in comparison to the instantaneous load, synthesized load (±n or +n) was much closer to the true load. In addition, the synthesized load (±n, +n) resulted in smaller variance, which indicated that some larger errors in the NN’s output were smoothed through the exploration strategy. For example, in Table 2, the variance of the exploration sequence with length 4 was smaller than that of length 2 (total: synthesized load (±2) vs. synthesized load (±1): 0.012 vs. 0.016, Table 2). In addition, the variance of an exploration sequence with length 3 was smaller than that of length 2 (total: synthesized load (+3) vs. synthesized load (+2): 0.014 vs. 0.017, Table 2). However, the synthesized load (±3) also increased the possibility of altering the synthesized estimation of external load away from the ground-truth load. Among the exploration sequences with same length (such as ±2 and +4, ±1 and +2), the forward inflation exploration strategy (such as +4, +2) could estimate the load with larger variances (±2 Antag-0 vs. +4 Antag-0: 0.003 vs. 0.005, Table 1).

**Figure 8:**
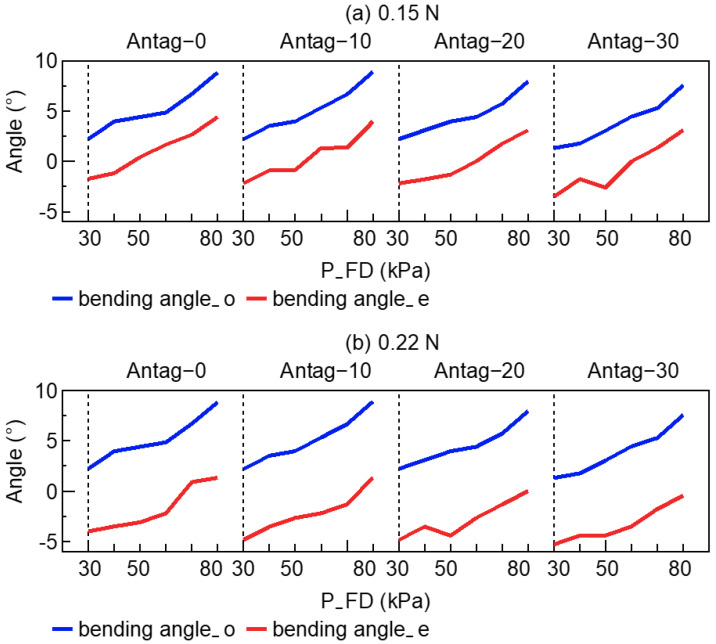
The bending angle_o and bending angle_e of the soft actuator (**a**) 0.15 N, (**b**) 0.22 N.

Ideally, different input sets (P_FD, P_FD, bending angle_o, bending angle_e) of the same external load should estimate almost the same instantaneous load through the trained NN. Figure 8 depicts the bending angle_o and bending angle_e for the network input. In the case of the identical P_FU and P_FD, the bending angles were regarded as the primary factors influencing the NN’s output. Figure 8b shows that in the early stage of 0.22 N, Antag-20, the disturbance problem caused the bending angle_e to have a fluctuation, resulting in a significant error in the network output (instantaneous load). Following the exploration strategy, the synthesized estimation of external load was smoother and steady.

**Figure 9:**
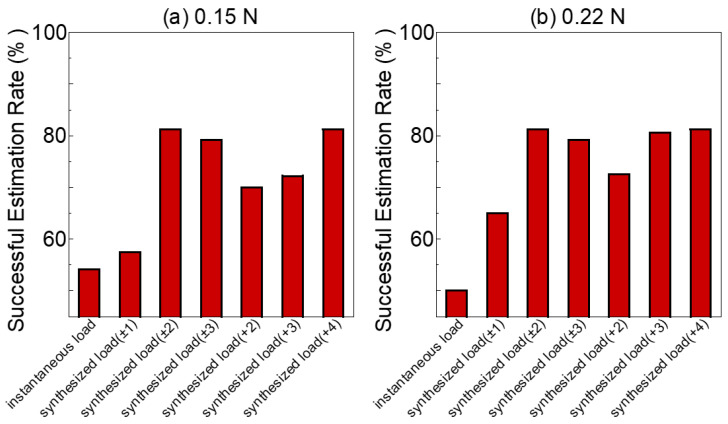
The successful estimation rate of the active load-sensing scheme with different sequence lengths (**a**) 0.15 N (**b**) 0.22 N.

Figure 9 summarizes the successful estimation rate for each exploration strategy. According to these results, relative to the instantaneous load, the successful estimation rate of each group after implementing an exploration strategy was enhanced to various degrees. However, a sequence that was too lengthy might introduce additional errors, while too short lengths would not be able to reduce the effect of errors; hence, it was crucial to select an appropriate exploration sequence. Because the ±2, ±3, and +4 exploration sequences had a successful estimation rate of 80% when estimating the two different external loads, the optimal exploration sequence appeared to be ±2.

**Figure 10:**
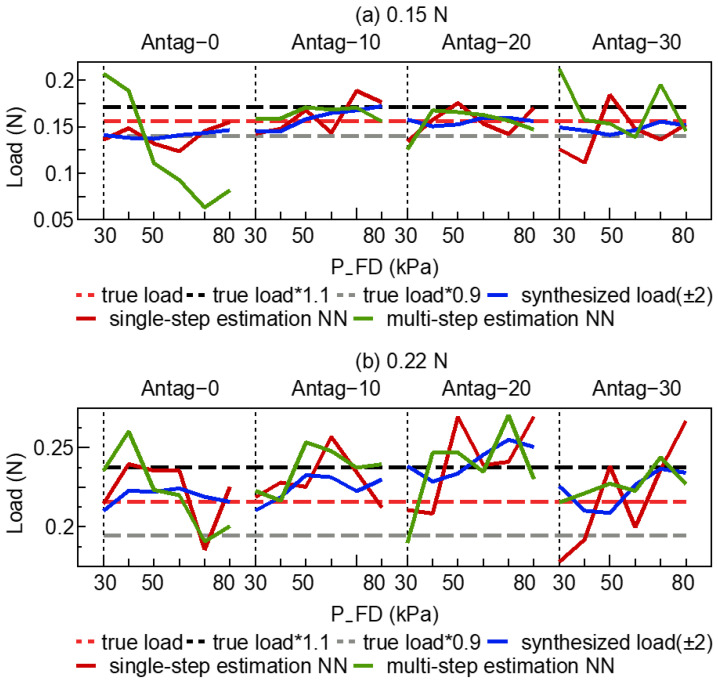
The test data of active load-sensing scheme with 5.0 mm endoscope from NN using single-step estimation NN and multi-step estimation NN (**a**) 0.15 N, (**b**) 0.22 N.

To confirm the effect of the optimal exploration sequence (±2), the input information of the optimal exploration sequence was concatenated as a total input vector to train another multi-step estimation NN, which means the multi-step estimation NN’s input size was (1, 16). The single-step estimation NN was same as the instantaneous load. Figure 10 shows the comparison of synthesized load (±2), single-step estimation NN and multi-step estimation NN. It was confirmed that individual points of multi-step estimation NN frequently deviated from the ground-truth value compared with the synthesized load (±2) and single-step estimation NN. For example, in 0.15 N, the average and variance of multi-step estimation NN (0.152 N, 0.0365) was larger than the single-step estimation NN (0.151 N, 0.0197) and synthesized load (±2) NN (0.150 N, 0.0195).

### 3.4. The Results of NN-Based Inverse Dynamics Model for Stiffness

**Figure 11:**
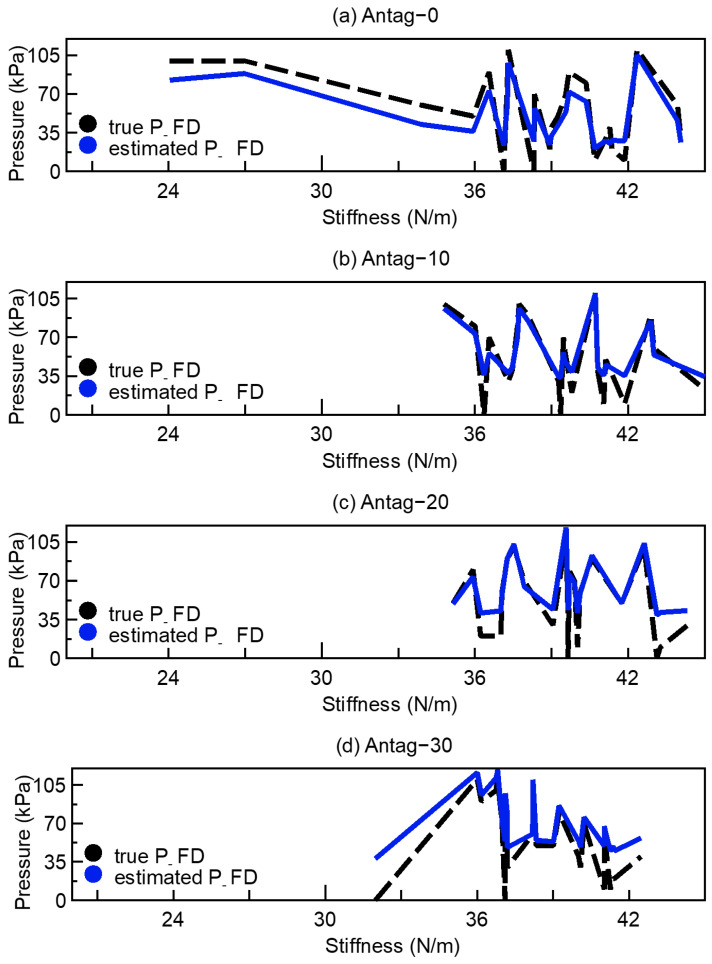
The test data of adjusting stiffness with 5.0 mm endoscope from NN (**a**) Antag−0, (**b**) Antag−10, (**c**) Antag−20, (**d**) Antag−30.

The NN-based inverse dynamics model for stiffness adjustment calculates the required P_FD when the desired stiffness was specified. The results are shown in Figure 11. As shown, even though the relationship between stiffness and air pressure was nonlinear, the trained inverse dynamics model was able to modify the air pressure value according to the desired stiffness. However, when the true P_FD was below 35 kPa, the air pressure could not be accurately predicted.

## 4. Discussion

The experiments were performed on the DoF 1 (composed of FU and an FD chamber) to further investigate the impact of various inflating circumstances on the soft actuator‘s stiffness and bending angle_o. As can be seen from Figure 4, the P_FU and P_FD had opposing effects on bending angle_o, and the P_FU chamber could play an inhibitory role on the bending angle_o, whereas the P_FD chamber increased the bending angle_o. The asymmetric deformation of the air chamber on both sides caused the bending of the soft actuator [33]. Larger asymmetric deformations may result in more extreme bending. A larger P_FD increased the symmetrical deformation of the soft actuator in response to the expansion of the FD, whereas the expansion of the FU decreased the asymmetric deformation, which was the major source of the inhibitory influence on the bending angle_o.

Nonetheless, this inhibitory effect was not permanent and even diminished when the P_FD increased (Figure 4a). Due to the strain-limiting cotton fibers on the outside of the soft actuator which limit the deformation of the air chamber to the exterior, the air chamber can only expand to the hollow structure inside of the soft actuator. As a result, the expanding FD may eventually interfere with the expansion of FU at the inside of the soft actuator, thereby increasing the asymmetric deformation and bending angle_o.

Meanwhile, based on the properties of the endoscope, we knew that the endoscope’s stiffness could change by changing the endoscope’s bending angle; in particular, the endoscope’s stiffness could be higher when the endoscope’s bending angle was very larger. The stiffness of the soft actuator also could be changed by changing the bending angle_o as shown in Figure 6a. This showed that the whole stiffness (endoscope + soft actuator) varied with different bending angle_o (Figure 6). Figure 5 demonstrates that the P_FU influenced the stiffness of the pneumatic actuator. In addition, the endoscope and the FU could squeeze each other when the chamber was expanded by air pressure, and the contact and friction forces between them were unstable. The friction force could inhibit the bending of the soft actuator and affect bending angle_o. All of the above could cause interference and make it difficult to estimate the external load. Since this effect was nonlinear and was dependent on both the P_FD and the endoscope’s diameter, developing a reliable analytical model was challenging. This nonlinear effect relied on input information of the active load-sensing scheme and was necessary to be found through NNs.

At high P_FD (over 90 kPa), the soft actuator almost exhibited significant stiffness (over 30 N/m) due to the silicon material of the air chamber being massively stretched and rendered impossible to deform under external loads [34]. In Figure 5c,d, Antag-0-e5.4 mm showed a lower stiffness than Antag-0-e5.0 mm. This was due to the fact that the stiffness of the 5.4 mm endoscope was lower than that of the 5.0 mm endoscope which mentioned in Section 2.1. The 5.4 mm endoscope should have been easier to bend by the soft actuator than the 5.0 mm endoscope. However, the larger diameter of endoscope could more significantly inhibit the expansion of the chamber and reduce the bending angle_o. Therefore, in Figure 4c,d, the bending angle_o of Antag-0-e5.0 mm was less than that of Antag-0-e5.4 mm.

To improve the accuracy of the external load estimation utilizing the active load-sensing system, an environment exploration strategy was presented and employed in a prototype experiment. Figure 7 and Figure 9 demonstrate that the proposed exploration strategy had substantially enhanced the external load estimation accuracy and the successful estimation rate. Combining Figure 7 and Figure 8, when the exploration strategy was not used and the input information (such as bending angle_e) produced certain perturbations, the end-to-end output of the NN also deteriorated. The inaccuracy between instantaneous load and true load could be averaged out by exploration, as shown in Figure 7 for Antag-30 with 50 kPa under 0.15 N and Antag-0 with 70 kPa under 0.22 N. The exploration strategy used the idea of a moving average to average the output error, making the final output smoother. This could ameliorate the problem that appears in related research [16] where individual points are frequently far from the true value.

Moreover, according to Table 1 and Table 2, the mean value of the synthesized estimation of external load almost increased as the length of the sequence increased, while the variance decreased. For instance, +4 had a larger mean error and a lower variance error than +2, whereas ±1 and ±3 were identical. This indicated that longer sequences resulted in reduced variance in estimation, and also increased the risk of estimation errors. Verification indicated that the mean value of the estimation load performs well and the successful estimation rate was highest for sequence lengths of ±2 and +4 (with the exception of the Antag-20 under 0.22 N group). Although ±2 and +4 had the same sequence length, ±2 inflates two steps forward and deflates two steps backward, and the maximum air pressure after exploration was 20 kPa higher/lower than the original air pressure. Meanwhile, +4 inflates forward. The final exploration air pressure was 40 kPa higher than the initial air pressure. Figure 4 demonstrates that the change in angle increased as air pressure rises, indicating that the change in angle of the ±2 sequence was lower than that of the +4 sequence, resulting in a shorter exploration time and greater efficiency. Moreover, Figure 2 indicates that the bending angle_o of Antag-20-e5.0 mm altered very little at 30–80 kPa of P_FD, which explains why the Antag-20 under 0.22 N group could not make a successful estimate. Thus, we hypothesized that when the difference between the two bending angles is small, the network’s load estimations would suffer. At the same time, the optimal exploration sequence (±2) did not have generalizability, and thus this optimal exploration sequence was based on the experiment results of our soft actuator and could not be presumed.

On the other hand, multi-step estimation NN was trained use all the information of the optimal exploration sequence as input. The results in Figure 10 show that this method could not improve the effect of the instantaneous load and could bring a larger error. The error of instantaneous load was due to the input information (such as bending angle_e, Figure 8) that produces a certain perturbation. When the input information was increased, the certain perturbation which was produced by input information was possible to be harnessed and made the instantaneous load far from the true label. Therefore, the optimal exploration sequence was not suitable to use as the input for the NN.

The NN-based inverse dynamics model could estimate appropriate P_FD for the desired stiffness, although only for pressures above 35 kPa. However, for pressures below 35 kPa, the model performs poorly, which might because when the P_FD was very low, the stiffness of the actuator varied very little, preventing the model from properly estimating the required P_FD.

The contributions of our study include:

1. In the related research [30], the NN generated very deviated predictions for the real test samples. In our work, we proposed an active load-sensing scheme and an environment exploration strategy to optimize the instantaneous load from NN. Moreover, we also investigated the optimal exploration sequence which could reduce the estimation error to less than 0.01 N and increase the successful estimation rate to 80%. The proposed method could not only help surgeons to select the suitable stiffness of the soft actuator based on the correct external load, but also provide the signal for engineering a better feedback controller design.
2. Furthermore, we also proposed an inverse dynamics model for stiffness, which could help surgeons easily adjust a suitable stiffness to resist different external loads during the MIS procedure and keep the surgical view stable. The proposed model could be used for in-depth study of soft actuators’ stiffness or impedance control in the future.

## 5. Conclusions and Future Works

In this paper, we proposed a NN-based active load-sensing scheme and stiffness adjustment for a soft actuator to provide a stable surgical view during MIS. To properly estimate the external load and thus determine an appropriate stiffness for the soft actuator, we further optimized the estimation results using an environment exploration strategy.

The results demonstrated that the exploration strategy had substantially enhanced the external load estimation accuracy and the successful estimation rate compared to the instantaneous estimation of NNs. The optimal exploration sequence was determined to be synthesized load (±2), with the highest successful estimation rate, smaller exploration range and shorter exploration time. An inverse dynamics model for stiffness adjustment based on a NN was proposed and implemented, with results demonstrating the model’s capacity to precisely calculate the air pressure P_FD for achieving the desired stiffness for the soft actuator.

Future studies include expansion of the active load-sensing scheme to different directions of multi-DoFs soft actuators and to design a closed-loop feedback controller for desired angle and stiffness.

## Figures and Tables

**Table 1:** The mean and variance of different exploration sequences with true load 0.15 N.

P_FU		True Load	Instantaneous Load	Synthesized Load (±1)	Synthesized Load (±2)	Synthesized Load (±3)	Synthesized Load (+2)	Synthesized Load (+3)	Synthesized Load (+4)
Antag-0	Mean (N)	0.156	0.140	0.141	0.141	0.141	0.143	0.144	0.144
	Variance (N^2^)	3.413 × 10^−5^	0.011	0.007	0.003	0.004	0.008	0.007	0.005
Antag-10	Mean (N)	0.156	0.161	0.159	0.159	0.159	0.166	0.167	0.168
	Variance (N^2^)	8.078 × 10^−6^	0.018	0.013	0.011	0.009	0.010	0.009	0.006
Antag-20	Mean (N)	0.156	0.156	0.155	0.156	0.155	0.158	0.157	0.156
	Variance (N^2^)	1.479 × 10^−5^	0.015	0.006	0.004	0.002	0.003	0.002	0.003
Antag-30	Mean (N)	0.156	0.143	0.145	0.148	0.149	0.149	0.151	0.150
	Variance (N^2^)	1.742 × 10^−5^	0.023	0.007	0.005	0.005	0.006	0.006	0.006
Total	Mean (N)	0.156	0.150	0.150	0.152	0.151	0.154	0.155	0.154
	Variance (N^2^)	4.367 × 10^−5^	0.020	0.009	0.008	0.009	0.010	0.011	0.010

**Table 2:** The mean and variance of different exploration sequences with true load 0.22 N.

P_FU		True load	Instantaneous Load	Synthesized Load (±1)	Synthesized Load (±2)	Synthesized Load (±3)	Synthesized Load (+2)	Synthesized Load (+3)	Synthesized Load (+4)
Antag-0	Mean (N)	0.216	0.223	0.221	0.219	0.218	0.221	0.220	0.219
	Variance (N^2^)	4.357 × 10^−5^	0.019	0.010	0.005	0.004	0.010	0.007	0.004
Antag-10	Mean (N)	0.216	0.229	0.225	0.224	0.223	0.227	0.228	0.228
	Variance (N^2^)	1.029 × 10^−5^	0.014	0.013	0.008	0.005	0.010	0.006	0.004
Antag-20	Mean (N)	0.216	0.240	0.239	0.242	0.241	0.247	0.247	0.246
	Variance (N^2^)	1.479 × 10^−5^	0.025	0.015	0.009	0.006	0.010	0.008	0.007
Antag-30	Mean (N)	0.216	0.218	0.221	0.224	0.226	0.228	0.230	0.230
	Variance (N^2^)	1.742 × 10^−5^	0.031	0.017	0.011	0.006	0.017	0.016	0.012
Total	Mean (N)	0.216	0.228	0.226	0.225	0.227	0.231	0.231	0.231
	Variance (N^2^)	1.134 × 10^−5^	0.025	0.016	0.012	0.013	0.017	0.014	0.013

## Data Availability

The data presented in this study are available on request from the corresponding author.

## Appendix A

Figure A1 displays the structure and characteristics of a three-DoFs antagonistic chamber pneumatic soft actuator with two DoFs (DoF 1 and DoF 2) at the front driven by four symmetrical pneumatic chambers (FU, FD, FL, FR) and one DoF (DoF 3) at the rear driven by two symmetrical pneumatic chambers (RU, RD). The purpose of DoF 1 and 2 is to finish specific surgical actions such as prostate probing, suturing and stripping. The two chambers (FU and FD, FL and FR) at each DoF constitute an antagonistic mechanism for regulating stiffness and bending. The function of DoF 3 can control the forward movement of the robot, enabling it to avoid impediments and pass organs.

An internal access channel comprised of a silicon tube (Dragonskin 30, Smooth-On, Inc., USA) was positioned in the center of the entire soft actuator (radius: 4.5 mm, length: 150 mm, thickness: 1 mm), accommodating flexible endoscopes with a diameter of less than 8 mm. Soft actuators were fabricated by 3D-printed molds using PLA (Ultimaker 2 Extended+, Ultimaker B.V., The Netherland). Dragonskin 10 MEDIUM (Smooth-On, Inc., USA) were used to fabricate the main body of the soft actuators. Dragonskin 10 MEDIUM comes in two parts that were mixed together at a 50:50 mass ratio. The mixture was subjected to a vacuum for a period of 15 min to de-air and was then slowly poured in the appropriate molds. Dragonskin 10 MEDIUM was used as the final coating to fix the strain-limiting cotton fibers (major radius: 8 mm, minor radius: 0.4 mm, axial pitch: 1 mm, number of turns: 70) with round restraint.
